# SynSAM: a hybrid synchronous learning framework with knowledge retention for prostate zonal segmentation leveraging the segment anything model

**DOI:** 10.1007/s11517-026-03522-2

**Published:** 2026-02-16

**Authors:** Chetana Krishnan, Ezinwanne Onuoha, Alex Hung, Kyung Hyun Sung, Harrison Kim

**Affiliations:** 1 Department of Biomedical Engineering, The Ohio State University, Columbus, OH 43210 USA; 2 Department of Radiology, The University of California, Los Angeles, Los Angeles, CA 90404 USA; 3 Department of Radiology, The Ohio State University, Suite 1255, 2050 Kenny Road, Columbus, OH 43221 USA

**Keywords:** Knowledge retention, Hybrid architecture, Transformer, Catastrophic forgetting, Prostate zonal segmentation

## Abstract

Accurate prostate zonal segmentation remains challenging due to domain shifts across institutions and the need for precise anatomical delineation. While transformer-based models like the Segment Anything Model (SAM) show promise, they struggle in medical domains, particularly with continual learning due to catastrophic forgetting. This study introduces SynESAM, a hybrid transformer-CNN segmentation framework that combines SAM’s image encoder with a CNN-based decoder. SynESAM employs synchronous learning strategies, specifically Elastic Weight Consolidation (EWC) and variational EWC (vEWC), to preserve prior knowledge during continual learning. The model was trained and evaluated on two datasets, UAB (in-house) and ProstateX (public), using Dice Similarity Coefficient (DSC) and Mean Surface Distance (MSD) as metrics. SynESAM significantly outperformed 16 benchmark models on both datasets. On UAB, it improved average DSC by 9.84% (TZ) and 18.54% (PZ), with worst-case DSC gains of 18.96% (TZ) and 31.53% (PZ). MSD improved by 40.93% (TZ) and 30.02% (PZ). In cross-dataset testing, SynESAM-EWC achieved up to 19.73% DSC improvement and 25.84% MSD reduction for PZ. SynESAM-vEWC showed slightly lower yet consistent gains. Overall, SynESAM provides robust, anatomically accurate segmentation across datasets by integrating transformer generalization, CNN efficiency, and continual learning strategies that mitigate catastrophic forgetting.

**Graphical Abstract:**

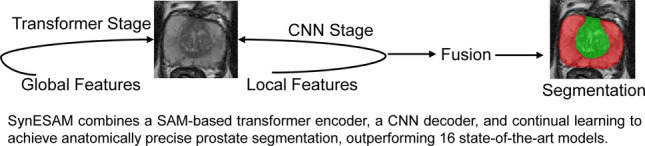

## Introduction

Prostate zonal segmentation plays a crucial role in biomarker quantification, serving as a fundamental step for the accurate assessment of prostate diseases, including benign prostatic hyperplasia and prostate cancer [1]. The differentiation of prostate zones, such as the peripheral zone (PZ) and transition zone (TZ), is essential for early diagnosis, treatment planning, and monitoring of disease progression [2]. Precise segmentation enables the improved extraction of radiomic features, facilitating the development of robust biomarkers for personalized treatment strategies [3]. Over the years, machine learning has revolutionized medical image analysis, enhancing both the efficiency and accuracy of segmentation tasks [4]. Traditional methods that relied on handcrafted features and manual delineation were time-consuming, prone to operator bias, and limited in reproducibility, often leading to high inter-observer variability [5]. Deep learning (DL), particularly convolutional neural networks (CNNs), has significantly advanced medical image segmentation by learning hierarchical feature representations directly from imaging data [6]. More recently, transformer-based architecture and hybrid models combining CNNs and transformers have demonstrated state-of-the-art performance in various medical imaging tasks [7].

A significant breakthrough in medical image segmentation has been the emergence of foundational models, which are pre-trained on large-scale datasets and designed to generalize across various domains [8]. Among these, the Segment Anything Model (SAM) has gained attention for its ability to perform promptable segmentation across diverse imaging modalities. SAM, developed by Meta AI, leverages a transformer-based architecture and extensive pretraining to enable zero-shot and few-shot segmentation, making it highly adaptable to new tasks with minimal labeled data [9]. While SAM has demonstrated remarkable generalizability, its direct application to medical imaging, particularly prostate zonal segmentation, remains challenging due to differences in imaging characteristics between natural and medical images. Furthermore, medical image segmentation often requires fine-grained anatomical delineation, which SAM, in its original form, may not fully capture without adaptation [10].

Another critical problem with models is catastrophic forgetting, which occurs when models are exposed to sequential learning tasks. Traditional deep learning architectures, including CNNs and transformers, are typically trained in a static manner, where they learn from a fixed dataset [11]. However, in real-world medical applications, models must continuously adapt to new data distributions, imaging modalities, and patient populations. When these models are trained on new datasets, they tend to overwrite previously learned knowledge, resulting in a significant drop in performance on earlier tasks, a phenomenon known as catastrophic forgetting [12].

This issue is particularly pronounced in longitudinal medical imaging studies and multi-institutional learning scenarios, where DL models must retain prior knowledge while adapting to new imaging conditions [13]. For example, in prostate zonal segmentation, variations in MRI scanners, acquisition protocols, patient demographics, and labeling standards between institutions create a major challenge for deep learning models. A model trained on data from one institution often exhibits significant performance degradation when tested on data from another institution, as it fails to generalize across domain shifts [14]. This phenomenon arises due to the model’s reliance on dataset-specific features, leading to domain bias that hinders its robustness in unseen environments [15]. Moreover, transformer and CNN models, while demonstrating remarkable generalization in natural image segmentation, are not inherently designed to retain knowledge over time or adapt incrementally to new medical datasets. As a result, applying a deep learning model directly to prostate zonal segmentation across different institutions may lead to inconsistent performance due to its lack of continual learning capabilities [16]. This necessitates adaptive learning strategies that not only enhance SAM’s segmentation capabilities but also ensure that knowledge gained from previous datasets is retained and leveraged for future learning tasks.

Another key challenge in medical image segmentation arises from the computational inefficiencies associated with using only transformer-based architectures [17]. Transformer models, while highly effective in capturing long-range dependencies and global context, require extensive computational resources, making them impractical for real-time clinical deployment, especially in resource-constrained environments [18]. Their quadratic complexity in self-attention mechanisms leads to high memory consumption, which is particularly problematic when processing high-resolution 3D medical images such as prostate MRI scans [19]. On the other hand, CNN-based architectures, despite their efficiency in local feature extraction and reduced computational overhead, often struggle with capturing global spatial relationships, which are crucial for segmenting anatomically complex regions, such as the prostate zones [20]. Additionally, CNNs tend to suffer from limited generalization across different imaging domains, as they rely heavily on local texture information, making them susceptible to variations in scanner settings and noise [21]. Given these limitations, neither approach alone is optimal for prostate zonal segmentation, highlighting the need for a hybrid model that leverages the strengths of both architectures while mitigating their individual weaknesses.

Previous studies have explored various adaptations of the SAM to enhance its performance in medical image segmentation. Previous studies have explored various adaptations of SAM to improve medical image segmentation. MedSAM, fine-tuned on over one million medical image–mask pairs across 11 modalities, demonstrated superior performance compared to U-Net [8]. EMedSAM reduced computational overhead while retaining accuracy [22]. Mazurowski et al*.* evaluated SAM’s zero-shot performance on multiple datasets and found that domain-specific adaptation was required [23]. SAM3D extended SAM to volumetric data, improving efficiency for 3D medical imaging [24]. SAM-Med2D applied domain-specific modifications to enhance accuracy across various imaging modalities [25], whereas SAM4MIS employed prompt-based tuning for medical segmentation [26]. He et al. benchmarked SAM on 12 public medical image datasets and concluded that SAM required fine-tuning to achieve accuracy comparable to specialized medical segmentation architectures distinct from SAM itself [27]. Si et al. showed that parameter-efficient fine-tuning strategies outperformed conventional ones [28]. Qiu et al. incorporated an Inception module to improve ultrasound endometrium segmentation [29]. Finally, Plug-and-Play SAM integrated SAM features with nnUNet to improve robustness across tasks [30].

Beyond SAM adaptations, continual learning strategies have been explored to mitigate catastrophic forgetting in medical image segmentation. González et al*.* introduced Lifelong nnU-Net, a model designed to sequentially learn new segmentation tasks without degrading previous performance [31]. Perkonigg et al*.* utilized dynamic memory networks to retain past knowledge while integrating new medical imaging data, addressing the challenge of evolving medical datasets [32]. Chen et al*.* developed a Low-Rank Mixture-of-Experts model that selectively updates network parameters for new segmentation tasks while preserving previous knowledge, reducing memory overhead [33]. Meanwhile, Continual Segment, proposed by Ji et al*.*, was designed to incrementally segment 143 whole-body organs in CT scans by employing a fixed encoder with progressively added decoders [34]. Verma et al*.* examined privacy-preserving continual learning, evaluating twelve deep learning methods for class-incremental segmentation of retinal diseases while ensuring patient data confidentiality [35]. Gautam et al*.* proposed Tf-GCZSL, a task-free generalized continual zero-shot learning approach, which enables continual learning in medical imaging settings where labels and tasks change over time [36].

In this manuscript, we introduce SynSAM, a hybrid synchronous learning framework that enhances SAM’s segmentation capabilities while improving efficiency and accuracy for prostate zonal segmentation. Current approaches face three key gaps: (1) SAM struggles in prostate MRI due to domain mismatch between natural and medical images, (2) CNN-based methods, while efficient, often fail to generalize robustly across institutions, and (3) continual learning has rarely been applied to SAM in medical imaging, limiting its ability to retain knowledge across datasets. SynSAM addresses these challenges by unifying transformer-driven global context modeling, CNN-based anatomical detail preservation, and synchronous learning strategies (EWC and vEWC) that mitigate catastrophic forgetting. Unlike prior SAM adaptations, our framework jointly optimizes time and memory efficiency while preserving knowledge, enabling robust and generalizable prostate zonal segmentation across diverse clinical datasets.

## Methods

### synSAM overview

**Fig. 1:**
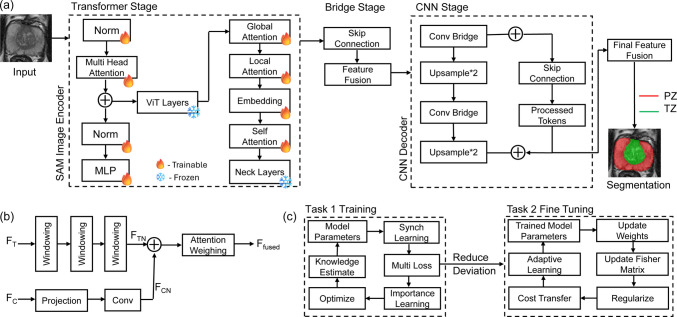
SynESAM pipeline and process. The proposed model, SynESAM, integrating SAM’s encoder and CNN decoder along with continual learning algorithms to preserve learned knowledge. (**a**) SynESAM Architecture, (**b**) Final Feature Fusion Pipeline, (**c**) Synchronous Learning Pipeline

We developed a hybrid Transformer-CNN architecture (synSAM) that combines the strengths of SAM-inspired transformers with a CNN architecture derived from a modified U-Net and ResNet. We explored two architectural variants, each integrating SAM’s components in different ways. SynESAM (Encoder-SAM Architecture) is one of these variants, where we utilized SAM’s image encoder as the backbone encoder to extract rich global features. At the same time, a modified UNet architecture served as the decoder to refine and reconstruct the segmentation mask. The combination of SAM’s transformer-based feature extraction and UNet’s CNN-based localization capabilities enables precise anatomical segmentation with improved boundary delineation. SynDSAM (Decoder-SAM Architecture) is the second variant, further explored in our ablation study, which utilizes ResNet-50 as the encoder to efficiently capture both local and hierarchical features. The modified mask decoder of SAM was used as the segmentation head. This approach leverages ResNet’s robust feature extraction with SAM’s powerful zero-shot segmentation capabilities, enabling a different but complementary method of integrating CNN and Transformer architectures. Figure 1a illustrates the overall architecture of SynESAM, as well as each of its components. The SynESAM architecture consists of three major stages: (1) the Transformer Stage, where SAM’s image encoder extracts high-dimensional features using multi-head attention and self-attention mechanisms; (2) the Bridge Stage, which applies global and local attention mechanisms along with feature fusion to refine embeddings and establish connections between the transformer and CNN components; and (3) the CNN Stage, where a modified U-Net-inspired decoder reconstructs the segmentation mask through convolutional bridges, upsampling layers, and skip connections, ensuring a balance between global context and local detail preservation.

### Transformer stage

The Transformer Stage in SynESAM is based on the SAM Image Encoder, which leverages a Vision Transformer (ViT)-based architecture for efficient global feature extraction. To improve segmentation accuracy while reducing training time, we employed transfer learning by utilizing pre-trained weights from the SAM image encoder. This enables the model to leverage rich, generalizable feature representations from SAM while allowing for domain-specific fine-tuning in prostate zonal segmentation. Most of the SAM’s layers were set to fully train to adapt to prostate MRI data, except for the ViT layers, as freezing these layers retains generalized feature extraction capabilities and prevents overfitting on our specific dataset. Assuming ${\uptheta }_{\mathrm{SAM}}$​ is the pre-trained weights of the SAM image encoder, we update only a subset of these weights, ${\uptheta}^{\prime}=\uplambda\;{\uptheta }_{\mathrm{SAM}}+\left(1-\uplambda \right){\uptheta }_{\mathrm{train}}$​ where ${\uptheta }_{\mathrm{train}}$ represents trainable weights, and $\uplambda \in \left[\mathrm{0,1}\right]$ is a scaling factor that determines how much of the pre-trained knowledge is retained versus fine-tuned. Since transformers operate on sequences, the input prostate MRI is first divided into non-overlapping image patches of size $p \times p$. Each patch is flattened and linearly projected into a D-dimensional embedding space $X=\left[{X}_{1},{X}_{2},\dots ,{X}_{N}\right],\hspace{1em}{X}_{i}\in {R}^{{p}^{2}}$ where $N=\frac{H\times W}{{p}^{2}}$​ is the total number of patches in an image of size $H \times W$, ${X}_{i}$​ represents the flattened pixel values of the ${i}^{th}$ patch. Each patch is transformed into an embedding vector via a linear projection: ${Z}_{0}=X{W}_{E}+{b}_{E},\hspace{1em}{Z}_{0}\in {R}^{N\times D}$ where ${W}_{E}\in {R}^{{p}^{2}\times D}$ is the learnable projection matrix, ${b}_{E}$​ is the bias term. To retain positional information, a learnable position encoding is added $Z={Z}_{0}+P,\hspace{1em}P\in {R}^{N\times D}$ where $P$ is the positional encoding matrix.

Once the patches are tokenized, they pass through a Multi-Head Self-Attention (MSA) mechanism, which enables the model to capture long-range dependencies in the prostate MRI. The attention function is computed as $A\left(Q,K,V\right)={\mathrm{softmax}}\left(\frac{Q{K}^{T}}{\sqrt{{d}_{k}}}\right)V$ where $Q=Z{W}_{Q}, K=Z{W}_{K} and V=Z{W}_{V}$ are learnable projections of the input embeddings and ${d}_{k}$​ is the dimensionality of the key vectors. After attention processing, the feature representations are passed through a multi-layer perceptron (MLP) block, which consists of two fully connected layers with a non-linearity ${Z}^{{\prime}{\prime}}={\mathrm{Norm}}\left({Z}^{\prime}+{\mathrm{MLP}}\left({Z}^{\prime}\right)\right)$ where ${\mathrm{MLP}}\left({Z}^{\prime}\right)=\upsigma \left({W}_{1}{Z}^{\prime}+{b}_{1}\right){W}_{2}+{b}_{2}M$ with ${W}_{1}$​ and ${W}_{2}$​ as trainable weight matrices, ${b}_{1}$​, ${b}_{2}$​ as bias terms, $\sigma$ representing a non-linear activation function (here GELU). The transformer stage further enhances feature extraction through additional attention mechanisms, including Global Attention, which captures long-range dependencies across the prostate MRI, Local Attention, which focuses on fine-grained spatial details relevant for segmentation, and Self-Attention, which refines internal feature representations before passing them to the CNN decoder. To smoothen the transformer-to-CNN transition, we introduce Neck Layers, which apply dimensionality reduction and feature normalization, ensuring that extracted representations are well-aligned for convolutional processing in the next stage as ${Z}_{\mathrm{neck}}={W}_{N}Z+{b}_{N}$ where ${W}_{N}$​ is a projection matrix, ${b}_{N}$​ is the bias term, ${Z}_{\mathrm{neck}}$​ is the final feature representation passed to the CNN decoder.

### Bridge stage

The Bridge Stage is a crucial transitional component in SynESAM, responsible for transforming feature representations from the SAM Image Encoder to the CNN-based decoder. Since transformers process images as tokenized sequences, whereas CNNs operate on spatial feature maps, this stage ensures that the long-range dependencies captured by the transformer are preserved while adapting the representations for convolutional processing. The Bridge Stage consists of two key operations: nested skip connections and feature fusion. To maintain multi-scale feature continuity between the encoder and decoder, we employ nested skip connections, which aggregate hierarchical features from different transformer layers. Standard skip connections only transmit features from a single layer, which can lead to information loss when transitioning from the transformer domain to the CNN domain. Instead, we design nested skip connections that aggregate outputs from multiple transformer layers, ensuring that both low-level fine-grained details and high-level contextual information are retained and are defined as ${S}^{\left(l\right)}={F}_{\mathrm{skip}}\left({X}^{\left(l\right)}\right)+{X}^{\left(l-1\right)}$ where ${S}^{\left(l\right)}$ represents the skip connection output at the layer $l$, ${X}^{\left(l\right)}$ is the current feature map, and ${X}^{\left(l-1\right)}$ is the previous skip connection output. The function ${F}_{\mathrm{skip}}$​ is a learnable transformation function, typically a $1 \times 1$ convolution or linear projection, which aligns feature dimensions between transformer outputs and CNN feature maps. To ensure multi-scale feature retention, we implement nested skip aggregation, where multiple skip connections from previous layers are weighted and summed. ${S}_{\mathrm{nested}}={\sum }_{i=1}^{N}{w}_{i}{S}^{\left(i\right)}$, where ${S}_{\mathrm{nested}}$​ is the final aggregated skip connection, and ${w}_{i}$​ is trainable weights that dynamically control the contribution of different layers. This nested mechanism ensures that hierarchical information from both shallow and deep transformer layers is transferred efficiently to the CNN decoder, preventing the loss of crucial anatomical features.

Once the hierarchical features are transferred via nested skip connections, the next step is feature fusion, which ensures smooth integration between transformer-based embeddings and convolutional feature maps. Since the transformer outputs high-dimensional embeddings, while CNNs process structured spatial feature maps, the fusion operation aligns the feature dimensions and enhances the spatial coherence of segmentation features. Feature fusion is mathematically formulated as ${F}_{\mathrm{fusion}}={W}_{f}{S}_{\mathrm{nested}}+{W}_{t}T$, where $={F}_{\mathrm{fusion}}$ is the final fused feature map, ${S}_{\mathrm{nested}}$ is the output from the nested skip connection, and $T$ is the final feature representation from the transformer neck layers. The ${W}_{f}$ and ${W}_{t}$ are learnable projection matrices that align the feature spaces before passing them to the CNN decoder. Through feature fusion, the long-range contextual information from transformers is combined with local spatial details, making it highly effective for prostate zonal segmentation. This step ensures that the decoder receives a rich feature representation that balances global contextual awareness with localized segmentation precision.

### CNN stage

The CNN Stage in SynESAM serves as the decoder, responsible for progressively reconstructing the segmentation mask by upsampling the refined feature representations received from the Bridge Stage. This stage is based on a modified U-Net architecture to ensure precise localization and boundary refinement of the prostate zones. Once the feature embeddings transition from the transformer domain to the CNN decoder, they pass through Convolutional Bridge Layers (CBL), which are responsible for refining feature representations for better segmentation performance, aligning feature dimensionality to match CNN-based processing, and enhancing spatial details lost during transformer-based encoding. Each CBL consists of a $3\times 3$ convolution followed by a non-linearity ${F}_{\mathrm{conv}}=\upsigma \left({W}_{c}*X+{b}_{c}\right)$ where ${W}_{c}$ is the convolution kernel, $X$ is the input feature map, ${b}_{c}$​ is the bias term, $\sigma$ is a non-linear activation function (GELU). This operation enhances spatial information, ensuring that fine-grained structures of the prostate regions are well-preserved before upsampling. The CBL is followed by progressive upsampling to reconstruct the spatial resolution of the segmentation mask. Since transformers operate at a lower resolution than the input image, the CNN decoder must restore the feature maps to the original resolution, defined as ${F}_{\mathrm{upsample}}={\mathrm{Upsample}}\left({F}_{\mathrm{conv}},{\mathrm{scale}}=2\right)$ where: ${F}_{\mathrm{conv}}$​ is the input feature map from the previous convolution, ${\mathrm{scale}}=2$ represents doubling the spatial resolution. We utilized a combination of bilinear interpolation and transposed convolution to achieve upsampling, allowing the network to recover fine details in the segmentation mask. To enhance feature propagation and retain hierarchical information, we implemented skip connections between the CNN decoder and processed tokens from the Bridge Stage. This allows the model to preserve both global transformer-based features and local CNN-based refinements.

### Final feature fusion

The Final Feature Fusion module, as seen in Fig. 1b, is the last processing stage before generating the segmentation mask. It effectively merges hierarchical information extracted by the transformer (global dependencies) and CNN (local spatial details) while ensuring that redundant or conflicting information is suppressed. Instead of direct concatenation, we employ structured feature transformation, attention weighting, and selective feature prioritization, enabling the fusion process to be dynamic and adaptive based on the learned significance of features. One major challenge in integrating transformer features is that they often contain high-dimensional, non-localized information that must be structured into spatially relevant groups before being merged with CNN outputs. Instead of treating all transformer features uniformly, we apply progressive windowing, where features are grouped into overlapping spatial blocks and processed hierarchically. Given a transformer output feature map, ${F}_{T}\in {R}^{{H}_{T}\times {W}_{T}\times {D}_{T}}$​, we introduced a recursive partitioning mechanism, ${F}_{T}^{\left(i+1\right)}={W}_{i}\cdot {F}_{T}^{\left(i\right)}$, where ${F}_{T}^{\left(i\right)}$ is the feature representation at the windowing level $i$, and ${W}_{i}$ is a learnable transformation matrix that progressively refines feature importance across windowing levels. Each level filters out less relevant features while amplifying region-specific patterns, ensuring that only the most critical transformer information is retained before fusion.

A direct fusion might lead to information misalignment and unstable optimization. To address this, we apply a dimensional calibration step before fusion, ensuring that both feature sources are in the same representational space. For the CNN feature map $F{F}_{C}\in {R}^{{H}_{C}\times {W}_{C}\times {D}_{C}},$ we first apply an adaptive projection ${F}_{CP}={W}_{P}{F}_{C}+{b}_{P}$​, where WPW_PWP​ is a projection matrix mapping CNN features into the transformer feature space, ${b}_{P}$​ is a bias term, and ${F}_{CP}$​ is the calibrated CNN feature map. Following this, we refine the feature distribution using a depth-wise convolution, ensuring that CNN-derived representations retain critical local dependencies while aligning with the transformer-based global descriptors, ${F}_{CN}=\upsigma \left({W}_{C}*{F}_{CP}+{b}_{C}\right)$, where ${W}_{C}$ is a learnable convolutional kernel, $*$ denotes depth-wise convolution, and $\sigma$ is an activation function (ReLU or GELU). This step ensures that CNN-derived features retain essential spatial hierarchies while making them compatible for fusion with transformer outputs.

Unlike conventional fusion techniques that equally merge CNN and transformer features, we introduce a context-aware prioritization mechanism that selectively weights features based on their relevance to the segmentation task. The intuition behind this approach is that some regions require stronger transformer-based contextual understanding (e.g., ambiguous boundaries), while others benefit more from CNN-based local refinements. To achieve this, we compute a feature relevance score for each spatial location using $$R\left(x,y\right)={\mathrm{softmax}}\left({W}_{R}\left[{F}_{T}\left(x,y\right)|{F}_{C}\left(x,y\right)\right]+{b}_{R}\right)$$, where $$R\left(x,y\right)$$ is the feature relevance score for pixel $$\left(x,y\right)$$, $${W}_{R}$$​ and $${b}_{R}$$​ are learnable parameters, and $$\;\;\!\!\!\!\mid\;\;\!\!\!\!$$ represents the concatenation of transformer and CNN features. Using this relevance score, we apply an adaptive scaling mechanism before the fusion, $${F}_{\mathrm{scaled}}\left(x,y\right)=$$$$R\left(x,y\right)\cdot {F}_{T}\left(x,y\right)+$$$$\left(1-R\left(x,y\right)\right)\cdot$$$${F}_{C}\left(x,y\right)$$.

This ensures that the final fused representation dynamically prioritizes the most informative source for each spatial location, enhancing segmentation precision. After feature prioritization, we apply an attention-guided feature selection mechanism, ensuring that salient features are amplified while redundant ones are suppressed. Instead of directly summing feature maps, we introduce learnable attention weights to determine the optimal contribution of each feature source as ${F}_{fused}=\alpha {F}_{T}+\beta {F}_{C}$ where $\alpha$ and $\beta$ are computed as $\alpha ,\beta ={\mathrm{softmax}}\left({W}_{A}{F}_{\mathrm{scaled}}+{b}_{A}\right)$. ${W}_{A}$​ and ${b}_{A}$ are learnable attention weights, the softmax function ensures that $\alpha +\beta =1$, maintaining adaptive balance. This step enhances feature selectivity, ensuring that noisy, less relevant features are suppressed while important global and local information is emphasized.

### Elastic weight consolidation (EWC)

EWC is a continual learning technique designed to prevent catastrophic forgetting, which occurs when a neural network forgets previously learned tasks while adapting to new ones [37]. EWC introduces a regularization term that prevents drastic changes to parameters that are critical for previous tasks by quantifying the importance of each parameter using the Fisher Information Matrix (FIM). Parameters that are more critical to the previous task receive stronger constraints, preventing them from drifting too far when learning new tasks. The EWC-regularized loss function is defined as ${\mathcal{L}}_{{\mathrm{EWC}}\left(\uptheta \right)}={\mathcal{L}}_{\mathrm{task}}\left(\uptheta \right)+\frac{\uplambda }{2}{\sum }_{i}{F}_{i}{\left({\uptheta }_{i}-{\uptheta }_{i}^{\mathrm{old}}\right)}^{2}$, where ${\mathcal{L}}_{\mathrm{task}}\left(\uptheta \right)$ is the loss function for the current task, and $\uplambda$ is the regularization strength, controlling how much past knowledge is preserved, ${F}_{i}$​ is the Fisher Information score, which measures the importance of parameter ${\uptheta }_{i}$​ to the previous task. ${\uptheta }_{i}^{\mathrm{old}}$​ is the parameter value learned from previous tasks. The term ${\left({\uptheta }_{i}-{\uptheta }_{i}^{\mathrm{old}}\right)}^{2}$ ensures that key parameters remain close to their original values. The Fisher Information Matrix (FIM) quantifies the sensitivity of the model’s loss function to changes in each parameter.

The Fisher score for each parameter is estimated as ${F}_{i}=E\left[{\left(\frac{\partial \mathrm{log}P\left(y|x,\uptheta \right)}{\partial {\uptheta }_{i}}\right)}^{2}\right]$, where $P\left(y|x,\uptheta \right)$ is the likelihood function of the model given the data $\left(x,y\right)$, $\frac{\partial \mathrm{log}P\left(y|x,\uptheta \right)}{\partial {\uptheta }_{i}}$​ represents the gradient of the log-likelihood with respect to ${\uptheta }_{i}$. ${F}_{i}$​ is computed as the expectation over the dataset. This Fisher matrix is estimated using a batch of training samples as ${F}_{i}\approx \frac{1}{N}{\sum }_{n=1}^{N}{\left(\frac{\partial \mathrm{log}P\left({y}_{n}|{x}_{n},\uptheta \right)}{\partial {\uptheta }_{i}}\right)}^{2}$, where *N* is the number of samples. The Fisher scores are stored along with the optimal parameters from previous tasks. When training on a new dataset, the EWC penalty is added to the loss function, preventing critical parameters from changing too much. When training a model without EWC, gradient updates freely modify all parameters. This can cause the model to lose important knowledge from previous datasets. The new loss function is $-\sum {\mathcal{L}}_{\mathrm{task}}\left(\theta \right)=-{\sum }_{n}n\mathrm{log}P\left({y}_{n}|{x}_{n},\theta \right)$, which does not consider previous tasks. With EWC, the model is restricted from making large changes to important parameters. The Fisher Information scores act as weights, ensuring that important parameters remain close to their previous values, ${\theta }_{i}^{new}$ = ${\theta }_{i}^{old}- \rho (\frac{\partial {L}_{EWC}}{\partial {\theta }_{i}})$, where ${\theta }_{i}^{new}$ is the updated parameter, $\rho i$ s the learning rate, and $\partial {L}_{EWC}$ is the gradient of the EWC-regularized loss. By scaling the gradient updates according to the Fisher score, EWC ensures that critical parameters do not change drastically, thereby preventing forgetting of previous segmentation knowledge.

### Variational elastic weight consolidation (vEWC)

vEWC improves upon standard EWC by modeling uncertainty in the estimation of parameter importance [38]. While standard EWC assumes that parameter importance is fixed and deterministic, vEWC introduces a probabilistic framework, allowing the model to dynamically adjust parameter constraints based on their relevance across multiple tasks. The key difference between vEWC and EWC is that vEWC models the posterior distribution of parameters rather than using a single Fisher Information estimate. This leads to a more adaptive and robust learning mechanism that retains knowledge better across sequential tasks. The variational form of EWC loss is defined as ${\mathcal{L}}_{\mathrm{vEWC}}\left(\uptheta \right)={\mathcal{L}}_{\mathrm{task}}\left(\uptheta \right)+\frac{\uplambda }{2}{\sum }_{\mathrm{i}}\left({\mathrm{F}}_{\mathrm{i}}+\mathrm{S}\left({\uptheta }_{\mathrm{i}}\right)\right){\left({\uptheta }_{\mathrm{i}}-{\uptheta }_{\mathrm{i}}^{\mathrm{old}}\right)}^{2}$, where ${\mathcal{L}}_{\mathrm{task}}\left(\uptheta \right)$ is the standard task loss (e.g., segmentation loss), $\uplambda$ is the regularization strength, ${\mathrm{F}}_{\mathrm{i}}$ is the Fisher Information score, $\mathrm{S}\left({\uptheta }_{\mathrm{i}}\right)$ is a stability function that dynamically computes additional parameter importance based on model behavior and $\left({\uptheta }_{\mathrm{i}}-{\uptheta }_{\mathrm{i}}^{\mathrm{old}}\right)$ ensures previously learned knowledge is not lost. Instead of using only Fisher Information to determine parameter importance, vEWC adds an uncertainty-based stability term, $\mathrm{S}\left({\uptheta }_{\mathrm{i}}\right)$, making it more flexible in adapting to new datasets while preserving old knowledge. Unlike Fisher Information (which is computed once per task), $S\left(\uptheta \right)$ is dynamically updated during training using parameter sensitivity analysis $S\left({\uptheta }_{i}\right)=\frac{1}{T}{\sum }_{t=1}^{T}\left|{\uptheta }_{i,t}-{\uptheta }_{i,t-1}\right|$.This tracks how much each parameter changes over time, ensuring that important parameters remain constrained even as new tasks are learned.

The importance of each parameter is now determined by $I\left({\uptheta }_{i}\right)={F}_{i}+S\left({\uptheta }_{i}\right)$, where $I\left({\uptheta }_{i}\right)$ is the total importance score of parameters ${\uptheta }_{i}$, computed as the sum of Fisher Information, *F*_*i*_, and Stability function, $S\left(\uptheta \right)$. If $S\left(\uptheta \right)$ is high, the model strongly restricts updates, preventing forgetting. If $S\left(\uptheta \right)$ is low, allowing updates, which enables adaptation to new tasks. The model learns new tasks while preserving important parameters from previous tasks using the updated loss function ${\mathcal{L}}_{{\mathrm{vEWC}}\left(\uptheta \right)}={\mathcal{L}}_{\mathrm{task}}\left(\uptheta \right)+\uplambda {\sum }_{i}I\left({\uptheta }_{i}\right){\left({\uptheta }_{i}-{\uptheta }_{i}^{\mathrm{old}}\right)}^{2}$. This ensures that only less important parameters are allowed to change freely while critical parameters remain stable.

### SynESAM with synchronous learning

As shown in Fig. 1c, the synchronous learning framework in SynESAM operationalizes continual learning by ensuring that knowledge preservation and adaptation coincide. In this setup, Task 1 training establishes the model’s initial segmentation capability while computing parameter importance. Task 2 fine-tuning refines performance on new data without catastrophic forgetting. In Task 1, model parameters are optimized using a segmentation loss and importance estimation, where the Fisher Information Matrix (FIM) and stability measures are computed to assess the criticality of each parameter. This importance estimation plays a key role in controlling future updates. During Task 2 fine-tuning, the model starts from previously trained parameters. Instead of unconstrained fine-tuning, adaptive learning ensures that only less critical parameters undergo significant changes while important ones are regularized. A key transition mechanism connects these phases, where the final learned parameters from Task 1 ${\theta }_{\mathrm{Task1}}^{*}$ serves as the initialization for Task 2 training, constrained by a stability-aware adaptation function ${\uptheta }_{\mathrm{Task2}}={\uptheta }_{\mathrm{Task1}}^{*}-\upeta \nabla \mathcal{L}{\mathrm{new}}\left(\uptheta \right)+\upgamma \nabla \mathcal{R}\left(\uptheta ,{\uptheta }_{\mathrm{Task1}}^{*}\right)$, where $\mathcal{L}{\mathrm{new}}$ represents the segmentation loss for the new dataset and $\mathcal{R}\left(\theta ,{\theta }_{\mathrm{Task1}}^{*}\right)$ is a regularization term that selectively controls how much deviation is allowed from the previous knowledge. The update ensures that the model does not diverge drastically from its previous learning while still adapting to new segmentation patterns. Throughout this process, the Fisher Matrix is updated, and adaptive learning mechanisms balance knowledge retention with flexibility, enabling multi-institutional generalization without requiring separate models for each dataset.

### Datasets

We trained and evaluated SynESAM using two datasets: an in-house prostate MRI dataset from the University of Alabama at Birmingham (UAB) and the publicly available ProstateX dataset [39].

#### UAB dataset

We trained and evaluated SynESAM on an in-house prostate mpMRI dataset acquired at the University of Alabama at Birmingham (UAB) [40]. Patients were eligible if they were 18 years or older and referred for mpMRI due to an elevated PSA or active surveillance. Exclusion criteria included standard MRI safety contraindications and renal failure. From November 2017 to April 2019, 71 patients were contacted and 44 participated (participation rate: 62%). The cohort had a median age of 65 years (range 50–77; 14 African Americans, 30 Caucasians). Within 72 days of mpMRI (mean ± SD: 22 ± 18 days), 25 patients (57%) underwent radical prostatectomy (*n* = 4) or biopsy (*n* = 21). Histopathology identified 31 prostate cancer lesions (19 in the peripheral zone, 12 in the transition zone), classified as low grade (Grade Group 1, *n* = 14), intermediate (GG 2–3, *n* = 10), and high grade (GG 4–5, *n* = 7). Tumor stages ranged from T1 to T3, with no nodal or distant metastases. Imaging was performed on a 3 T Siemens Prisma scanner with a turbo spin-echo T2-weighted sequence. Of the 44 cases, 34 were used for training and 10 for evaluation. The transition zone (TZ) and peripheral zone (PZ) were manually delineated by Dr. Harrison Kim, the principal investigator of the study, based on tissue contrast observed in T2-weighted MR images.

#### ProstateX dataset

We also used the publicly available ProstateX dataset, originally collected at Radboud University Medical Center and later released through the PROSTATEx Challenges [41]. This cohort comprises 204 patients (median age, 66 years; range, 48–83) with a median PSA of 13 ng/mL (range, 1–56). Each patient underwent mpMRI on Siemens 3 T systems (MAGNETOM Trio and Skyra). Scans included T2-weighted imaging (0.5 mm in-plane resolution), dynamic contrast-enhanced (DCE) imaging (1.5 × 1.5 × 4 mm^3^, temporal resolution 3.5 s), and diffusion-weighted imaging (2 × 2 × 3.6 mm^3^, three b-values: 50, 400, 800 s/mm^2^). Lesions were assigned a PI-RADS score of 3 or higher and verified with MRI-guided biopsy, then graded by an expert pathologist. The Gleason Grade Group distribution was 32% (GG1), 36% (GG2), 16% (GG3), 9% (GG4), and 7% (GG5). For model development, 183 cases were used for training and 21 for testing. While the UAB dataset includes a relatively small cohort of 44 patients, its integration alongside the larger ProstateX dataset highlights SynESAM’s ability to achieve cross-institutional robustness even with limited data availability, reflecting real-world clinical constraints where institution-specific datasets are often modest in size.

### Datasets preprocessing

All images used in this study were single-channel grayscale scans, originally formatted as $n\times 320\times 320$ (d × w × h), where $n$ represents the number of slices per scan. To ensure the most relevant prostate regions were retained, we applied center cropping, a method that extracts a focused region from the image by selecting a central portion. Instead of using fixed dimensions, we determined the cropping coordinates dynamically by dividing the image width and height by a predefined ratio, ensuring that the extracted region was adaptive to variations in prostate positioning. This method differs from conventional center cropping, which relies on subtracting half the crop size from the center; instead, it adjusts based on prostate distribution patterns. Following cropping, the images were resized to $20\times 128\times 128$ (depth × width × height) and normalized within the 0–255 intensity range to ensure uniformity across datasets. The 3D volumes were sliced for 2D segmentation. For segmentation, a multi-class approach was implemented, where the background was labeled as class 0, while the TZ and PZ were assigned class 1 and class 2, respectively. Additionally, random flipping and rotation augmentations were applied to increase dataset variability, effectively doubling the number of training samples and improving model generalization.

### Training implementation

The SynESAM model was trained, with ViT and neck layers of the SAM image encoder frozen, while all other components remained trainable to allow task-specific adaptation. The CNN stage consisted of four encoder blocks and four decoder blocks, providing a deep feature extraction and reconstruction pathway. The model was trained for 200 epochs using an initial learning rate of 1e-3, which was adjusted dynamically through learning rate decay based on improvements in validation loss. A patience level was set to prevent unnecessary training iterations if the model exhibited stagnation. For optimization, we employed the Adam optimizer, which was selected through a grid search with a batch size of 8 to strike a balance between computational efficiency and performance. The model was trained on a single NVIDIA GeForce RTX 3090 GPU, utilizing mixed-precision training to optimize memory usage and computational speed. The loss function was a combination of Dice Loss and Mean Squared Error (MSE) Loss, ensuring both segmentation accuracy and smooth learning of anatomical boundaries.

Additionally, gradient clipping was applied to stabilize training and prevent vanishing or exploding gradients. The training was conducted using PyTorch, with data loading and augmentation performed via the Torchvision library. To improve model generalization, on-the-fly data augmentation techniques were applied, including random flipping, rotation, and intensity normalization, ensuring robustness against variations in MRI acquisition protocols. The model’s performance was evaluated using the Dice Similarity Coefficient (DSC) and Mean Intersection over Union (mIoU), with the best-performing model weights saved based on improvements in the validation DSC.

For synchronous learning, SynESAM was first trained on one dataset and then fine-tuned and tested on another dataset to evaluate cross-domain generalization. The fine-tuning phase incorporated synchronized learning techniques, leveraging EWC or vEWC to prevent catastrophic forgetting while adapting to new datasets. The model was fine-tuned using only 15 training samples for 10 epochs, with a very low learning rate, to ensure stable parameter adaptation without overfitting. The same training configuration as the initial training phase was maintained, including batch size, optimizer, and hardware setup, with the addition of a continual learning loss component. The final loss function combined Dice Loss, MSE Loss, and synchronized learning constraints to balance new learning with the retention of previously acquired knowledge in segmentation. Early stopping was enabled to prevent overfitting on the limited fine-tuning dataset, and data augmentation was applied conservatively to maintain structural integrity in the small dataset.

### Experiments

We compared SynESAM’s performance with the 16 popular and published architectures for semantic segmentation. The performance evaluation consisted of two stages: an evaluation of performance architecture-wise and an evaluation of multi-institutional (knowledge retention) performance capability. The comparative models were Axial Attention (AxialAttn) [42], Dual Attention (DualAttn) [43], Efficient Channel Attention (ECA) [44], GCNet [45], Gaussian Context Transformer (GCT) [46], Gated Channel Transformation (GCT CVPR) [47], Linear Context Transformer (LCT) [48], r2Attn UNet [49], Triple Attention (tripelAttn) [50], Gated Attention (Gated Attn) [51], CAT, CSAM, GLCSA [52], DANet [53], MedFormer [54], and SwinUNeTR [55] networks. All the models were trained in the same manner as SynESAM, without the synchronous learning framework. All the models were evaluated using the Dice Similarity Coefficient (DSC) and Mean Surface Distance (MSD) on the unseen test set. The DSC is a widely used statistical metric for evaluating the spatial overlap between two binary segmentation masks, typically the *predicted segmentation* P and the *ground truth* G. It quantifies how similar the two regions are, providing a value between 0 and 1, where 1 indicates perfect agreement and 0 indicates no overlap. Mathematically, the Dice coefficient is defined as ${\mathrm{DSC}}=\frac{2\left|P\cap G\right|}{\left|P\right|+\left|G\right|}$ where ∣P∣ and ∣G∣ denote the number of voxels (or pixels) in the predicted and ground truth segmentations, respectively, and $\left|P\cap G\right|$ represents the number of voxels common to both. In practice, the DSC is computed by summing the intersection of the binary masks and normalizing by the total number of voxels in both masks. A higher DSC indicates a closer correspondence between the prediction and the ground truth, making it a standard performance indicator in medical image segmentation tasks. This measure is susceptible to both false positives and false negatives, ensuring that models are evaluated on their ability to delineate anatomical boundaries accurately.

MSD quantifies the average distance between the predicted segmentation surface and the ground-truth surface, providing a boundary-based measure of accuracy. Unlike region overlap metrics such as DSC, MSD directly evaluates how closely the model’s predicted boundaries follow expert annotations, which is particularly important for assessing the clinical reliability of prostate zonal segmentation. Formally, let $${S}_{P}$$​ denote the set of surface points on the predicted segmentation and $${S}_{G}$$​ denote the set of surface points on the ground truth. The directed average surface distance from $${S}_{P}$$​​ to $${S}_{G}$$​ is $$d\left({S}_{P},{S}_{G}\right)=$$$$\frac{1}{\left|{S}_{P}\right|}$$$${\sum }_{p\in {S}_{P}}$$$$\underset{g\in {S}_{G}}{\mathrm{min}}$$$$|p-g{|}_{2}$$. The MSD is then defined symmetrically as, $${\mathrm{MSD}}\left({S}_{P},{S}_{G}\right)=\frac{1}{2}\left[d\left({S}_{P},{S}_{G}\right)+d\left({S}_{G},{S}_{P}\right)\right]$$, where $$|p-g{|}_{2}$$ is the Euclidean distance between a predicted surface point $$p$$ and its closest ground-truth point $$g$$.

## Results

Tables 1 and 2 summarize the performance metrics of the proposed SynESAM model compared to other state-of-the-art methods on the UAB and ProstateX datasets, respectively. SynESAM demonstrated consistent improvements in prostate zonal segmentation performance across both datasets. On the UAB dataset, SynESAM achieved substantial gains, with average DSC improvements of 9.84% for the transition zone (TZ) and 18.54% for the peripheral zone (PZ). Notably, the model significantly improved worst-case DSC by 18.96% for TZ and 31.53% for PZ. In terms of MSD, SynESAM reduced errors by 40.93% for TZ and 30.02% for PZ, indicating superior boundary accuracy and segmentation consistency. On the ProstateX dataset, although the improvements were slightly more modest, SynESAM still outperformed the baselines with average DSC gains of 4.66% (TZ) and 11.23% (PZ). While the worst-case DSC for TZ decreased by 9.96%, the PZ saw a significant improvement of 29.88%. MSD also showed meaningful reductions: 4.43% for TZ and 19.47% for PZ, confirming SynESAM’s robustness across varying dataset characteristics.

**Table 1:** SynESAM performance metrics compared with nine other models on UAB test data

Model	Average Test DSC		Worst Case DSC		Average Test MSD	
	TZ	PZ	TZ	PZ	TZ	PZ
AxialAttn	0.85 ± 0.06	0.64 ± 0.11	0.73	0.47	1.63 ± 0.60	1.46 ± 0.34
DualAttn	0.90 ± 0.03	0.65 ± 0.08	0.83	0.56	1.02 ± 0.27	1.96 ± 0.70
ECA	0.89 ± 0.02	0.66 ± 0.10	0.85	0.51	1.13 ± 0.35	1.53 ± 0.37
GC	0.89 ± 0.03	0.65 ± 0.10	0.85	0.51	1.18 ± 0.35	1.58 ± 0.41
GCT	0.87 ± 0.04	0.63 ± 0.09	0.87	0.63	1.39 ± 0.43	1.60 ± 0.33
GCT CVPR	0.88 ± 0.02	0.65 ± 0.08	0.85	0.55	1.17 ± 0.34	1.92 ± 0.49
LCT	0.86 ± 0.05	0.62 ± 0.08	0.74	0.52	1.37 ± 0.48	1.73 ± 0.29
r2 Attn UNet	0.62 ± 0.10	0.51 ± 0.10	0.49	0.32	5.05 ± 1.54	3.00 ± 0.51
tripleAttn	0.87 ± 0.03	0.64 ± 0.09	0.80	0.52	1.36 ± 0.35	1.85 ± 0.45
SynESAM (Proposed)	**0.92 ± 0.01**	**0.74 ± 0.06**	**0.90**	**0.65**	**0.81 ± 0.16**	**1.24 ± 0.37**

Average Dice Similarity Coefficient (DSC), worst-case DSC and average Mean Surface Distance (MSD) of the proposed model (SynESAM) in comparison with popular networks for segmenting the peripheral (PZ) and transition (TZ) prostate zones on the unseen UAB data test set

**Table 2:** SynESAM performance metrics compared with nine other models on ProstateX test data

Model	Average Test DSC		Worst Case DSC		Average Test MSD	
	TZ	PZ	TZ	PZ	TZ	PZ
AxialAttn	0.76 ± 0.11	0.65 ± 0.11	0.36	0.47	1.58 ± 0.27	1.60 ± 0.65
DualAttn	0.81 ± 0.06	0.70 ± 0.07	**0.62**	0.54	**1.51 ± 0.61**	1.27 ± 0.36
ECA	0.78 ± 0.08	0.67 ± 0.09	0.48	0.44	1.83 ± 0.39	1.49 ± 0.44
GC	0.79 ± 0.08	0.63 ± 0.10	0.51	0.37	1.76 ± 0.39	1.57 ± 0.44
GCT	0.79 ± 0.08	0.63 ± 0.10	0.48	0.44	1.82 ± 0.40	1.72 ± 0.61
GCT CVPR	0.81 ± 0.08	0.69 ± 0.09	0.51	0.49	1.66 ± 0.39	1.27 ± 0.38
LCT	0.80 ± 0.06	0.69 ± 0.09	0.58	0.47	1.79 ± 0.50	1.33 ± 0.49
r2 Attn UNet	0.81 ± 0.09	0.71 ± 0.08	0.49	0.54	1.70 ± 0.59	1.48 ± 0.63
tripleAttn	0.79 ± 0.08	0.63 ± 0.10	0.46	0.39	1.76 ± 0.31	1.60 ± 0.46
SynESAM (Proposed)	**0.83 ± 0.10**	**0.74 ± 0.07**	0.44	**0.59**	1.63 ± 0.58	**1.18 ± 0.41**

Average Dice Similarity Coefficient (DSC), worst-case DSC and average Mean Surface Distance (MSD) of the proposed model (SynESAM) in comparison with popular networks for segmenting the peripheral (PZ) and transition (TZ) prostate zones on the unseen ProstateX data test set

Among the 16 evaluated models, those not included in Tables 1 and 2 yielded comparable DSC and MSD metrics to SynESAM. These models were instead used to benchmark the performance of the proposed framework under continual learning conditions, as shown in Table 3. SynESAM was further evaluated in a continual learning setting using two regularization strategies: Elastic Weight Consolidation (EWC; SynESAM-E) and its variant with reduced regularization strength (vEWC; SynESAM-VE). The experimental design focused on cross-institutional domain adaptation, wherein models were initially trained on one dataset and subsequently fine-tuned and evaluated on the other. Two transfer scenarios were tested: training on UAB and then fine-tuning on ProstateX (UAB ➔ ProstateX) and training on ProstateX and then fine-tuning on UAB (ProstateX ➔ UAB).

**Table 3:** Proposed model multi-institutional performance metrics compared with seven other models

Model	Region	Test Average DSC		Test Average MSD	
		UAB → ProstateX	ProstateX → UAB	UAB → ProstateX	ProstateX → UAB
Gated Attn	TZ	0.72 ± 0.09	0.85 ± 0.02	1.96 ± 0.48	**1.49 ± 0.34**
	PZ	0.61 ± 0.12	0.53 ± 0.14	2.14 ± 1.30	1.68 ± 0.43
CAT	TZ	0.57 ± 0.13	0.71 ± 0.07	2.52 ± 0.39	2.62 ± 0.51
	PZ	0.47 ± 0.11	0.43 ± 0.21	3.02 ± 1.46	2.66 ± 0.96
CSAM	TZ	0.66 ± 0.09	0.78 ± 0.06	2.20 ± 0.36	1.78 ± 0.49
	PZ	0.54 ± 0.10	0.56 ± 0.12	2.17 ± 0.58	2.06 ± 0.77
DANet	TZ	0.68 ± 0.11	0.84 ± 0.03	2.19 ± 0.66	1.84 ± 0.44
	PZ	0.59 ± 0.13	0.57 ± 0.13	2.34 ± 1.55	1.61 ± 0.51
GLCSA	TZ	0.59 ± 0.13	0.77 ± 0.12	2.06 ± 0.56	2.15 ± 1.15
	PZ	0.44 ± 0.12	0.42 ± 0.10	2.68 ± 0.98	2.80 ± 0.93
MedFormer	TZ	0.63 ± 0.11	0.79 ± 0.05	2.68 ± 0.71	2.20 ± 0.63
	PZ	0.57 ± 0.11	0.52 ± 0.17	2.45 ± 0.85	2.20 ± 0.57
SwinUNeTR	TZ	0.70 ± 0.09	0.78 ± 0.05	2.14 ± 0.48	1.91 ± 0.51
	PZ	0.57 ± 0.10	0.49 ± 0.20	2.23 ± 0.67	2.06 ± 0.77
SynESAM-VE	TZ	0.73 ± 0.09	0.83 ± 0.03	**1.68 ± 0.31**	1.87 ± 0.50
	PZ	0.60 ± 0.11	0.59 ± 0.14	1.81 ± 0.87	1.66 ± 0.44
SynESAM-E	TZ	**0.74 ± 0.09**	**0.85 ± 0.03**	1.70 ± 0.29	1.79 ± 0.49
	PZ	**0.64 ± 0.10**	**0.61 ± 0.13**	**1.78 ± 1.15**	**1.51 ± 0.42**

Average Dice Similarity Coefficient (DSC), and average Mean Surface Distance (MSD) of the proposed model with two learning techniques – EWC (SynESAM-E) and vEWC (SynESAM-VE) in comparison with popular networks when trained on UAB data and fine-tuned, tested on ProstateX data (UAB → ProstateX) and vice versa (ProstateX → UAB) for segmenting the peripheral (PZ) and transition (TZ) prostate zones

In the (UAB ➔ ProstateX) setting, SynESAM-E achieved significant improvements over baseline approaches, with average DSC increases of 14.59% (TZ) and 19.73% (PZ), and MSD reductions of 23.64% (TZ) and 25.84% (PZ). These results highlight SynESAM-E’s capacity to adapt to new domains while preserving anatomical precision. Similarly, in the (ProstateX ➔ UAB) setting, SynESAM-E maintained its effectiveness, yielding DSC gains of 8.12% (TZ) and 22.85% (PZ), alongside MSD reductions of 7.91% (TZ) and 27.20% (PZ). These findings underscore its robustness in retaining learned knowledge while adapting to unseen clinical data. SynESAM-VE, employing a more relaxed regularization constraint, followed a similar trend with slightly attenuated performance. In the (UAB ➔ ProstateX) configuration, SynESAM-VE improved DSC by 13.04% (TZ) and 12.25% (PZ), while MSD was reduced by 24.54% (TZ) and 24.59% (PZ). In the (ProstateX ➔ UAB) scenario, the model achieved DSC gains of 5.58% (TZ) and 18.82% (PZ) and MSD reductions of 3.79% (TZ) and 19.97% (PZ).

Statistical significance testing using two-tailed independent t-tests confirmed the superiority of SynESAM across both single- and cross-institutional evaluations. On the UAB dataset, SynESAM achieved significantly higher average DSC than all baseline models for both TZ (*p* < 0.001) and PZ (*p* < 0.001), with worst-case DSC improvements also highly significant (TZ and PZ, *p* < 0.001). Average MSD values were likewise significantly lower for SynESAM in both zones (*p* < 0.001). On the ProstateX dataset, SynESAM again outperformed all competing models in terms of average DSC (*p* < 0.001) and MSD (*p* < 0.001). For worst-case DSC, SynESAM demonstrated significant gains over all models except DualAttn, which achieved slightly higher TZ worst-case DSC (0.62 *vs*. 0.44, *p* ≈ 1.0). In the few-shot multi-institutional setting, SynESAM-E exhibited consistent statistical advantages: when transferring from UAB ➔ ProstateX, it significantly outperformed all models in both DSC (*p* < 0.001) and MSD (*p* < 0.001). When transferring from ProstateX ➔ UAB, SynESAM-E maintained significant improvements over nearly all models (DSC TZ: *p* < 0.001; DSC PZ: *p* < 0.001; MSD TZ: *p* < 0.001; MSD PZ: *p* < 0.001), performing comparably only to GatedAttn in TZ and to SynESAM-VE in PZ. Collectively, these results highlight SynESAM’s robustness and statistically significant superiority under rigorous two-tailed testing in both within-dataset and cross-institutional scenarios.

Overall, these results affirm SynESAM’s effectiveness in both standard and continual learning settings. The EWC-regularized variant, SynESAM-E, consistently delivered the best performance in terms of segmentation accuracy and boundary delineation. Its adaptability across heterogeneous clinical datasets makes it a promising solution for robust, scalable, and adaptive medical image segmentation in real-world applications.

## Discussion

### Prostate zonal segmentation performance of SynESAM

**Fig. 2:**
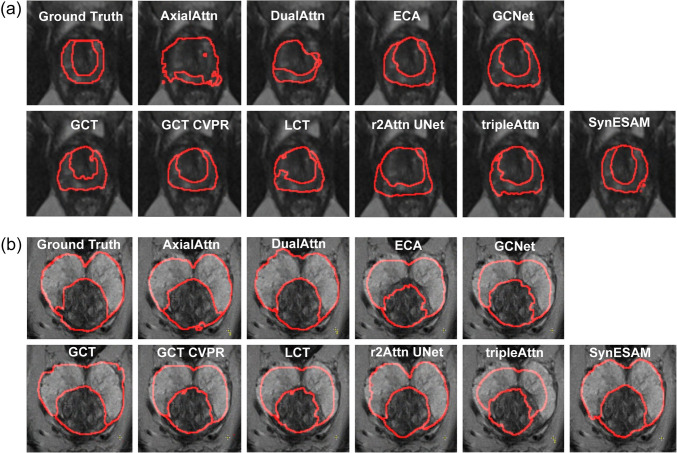
The best-case representative prostate images with peripheral and transition zones determined by SynESAM and nine other models. (a, b) The best-case representative prostate images with the boundaries of peripheral and transition zones determined manually (ground truth) or by SynESAM, AxialAttn, DualAttn, ECA, GCNet, GCT, GCT CVPR, LCT, r2Attn UNet, and tripleAttn, when (**a**) UAB or (**b**) ProstateX test datasets were employed

The SynESAM model demonstrated superior performance in prostate zonal segmentation compared to other competing architectures. As shown in Fig. 2, which outlines segmentation boundaries in red, SynESAM closely aligns with the ground truth in both the UAB (Fig. 2a) and ProstateX (Fig. 2b) datasets. Unlike other models, SynESAM preserves smooth, anatomically coherent boundaries and exhibits strong agreement with expert annotations. In the UAB dataset (Fig. 2a), models such as tripleAttn and r2Attn UNet exhibited notable segmentation errors, including over-segmentation and missed regions, resulting in structural distortions. SynESAM, in contrast, precisely delineated the prostate contours, preserving anatomical accuracy. This performance is attributed to its hybrid transformer-CNN architecture, which combines global contextual modeling from transformers with the spatial localization ability of CNNs.

Similar observations were made in the ProstateX dataset (Fig. 2b), where models such as DualAttn, ECA, and GCNet failed to consistently capture the true shape and position of both the transitional zone (TZ) and the peripheral zone (PZ). These models often produced irregular or poorly aligned boundaries, especially in the PZ, an area known for its challenging segmentation due to low contrast and structural complexity. SynESAM, however, produced smooth and continuous contours that accurately separated TZ from PZ and retained fine anatomical details, especially in the smaller PZ region, where other models typically exhibited under-segmentation or boundary blurring.

**Fig. 3:**
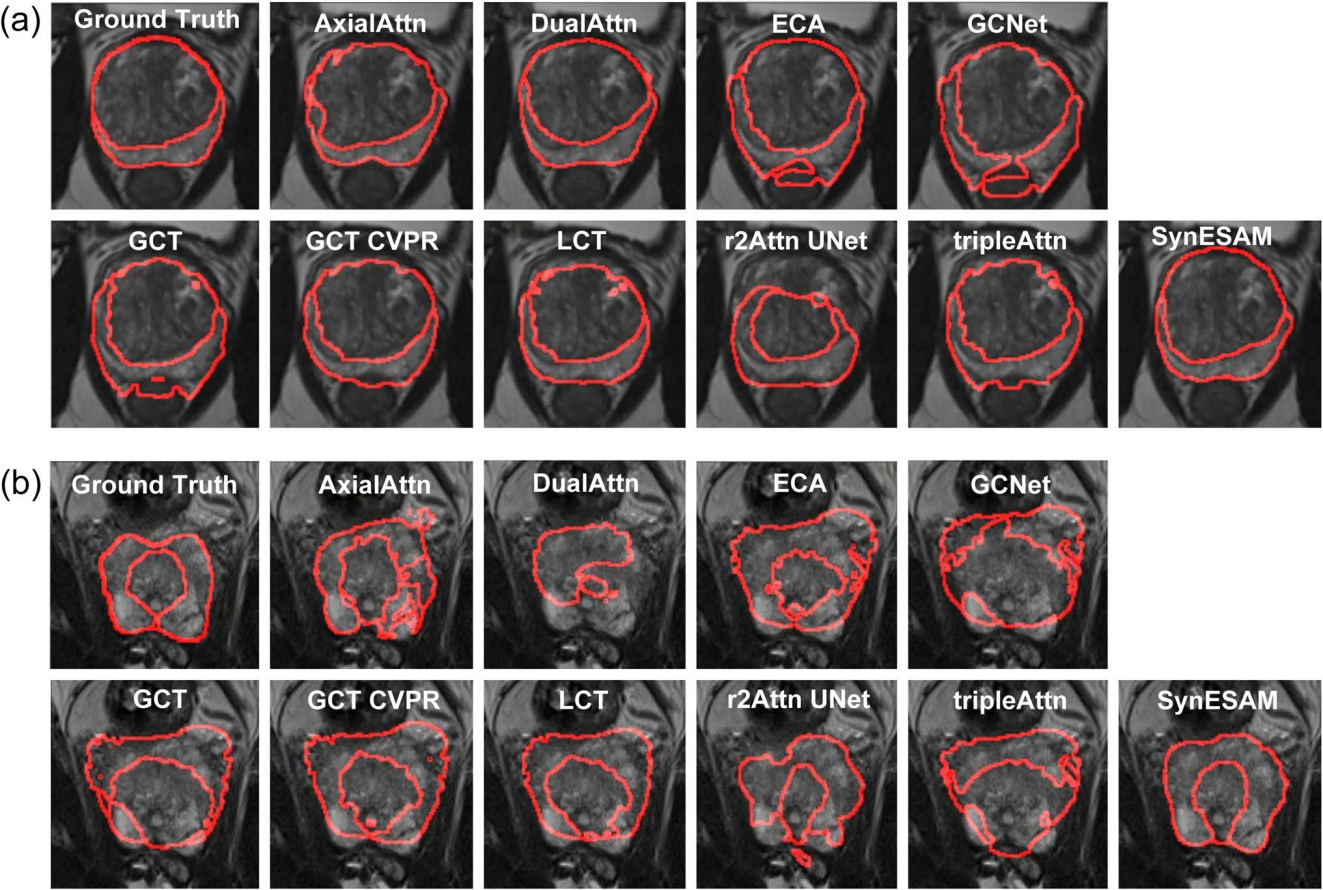
The worst-case representative prostate images with peripheral and transition zones determined by SynESAM and nine other models. (a, b) The worst-case representative prostate images with the boundaries of peripheral and transition zones determined manually (ground truth) or by SynESAM, AxialAttn, DualAttn, ECA, GCNet, GCT, GCT CVPR, LCT, r2Attn UNet, and tripleAttn, when (**a**) UAB or (**b**) ProstateX test datasets were employed

In Fig. 3, which further evaluates segmentation under more difficult conditions, SynESAM again outperformed competing methods. In Fig. 3a (UAB dataset), many models generated disjointed or fragmented boundaries that deviated from the ground truth. For instance, ECA and DualAttn models frequently exhibited over-segmentation, extending beyond the prostate region. SynESAM preserved boundary coherence and maintained a consistent separation between TZ and PZ without introducing noticeable distortions. Its segmentations were closed, continuous, and anatomically plausible.

Figure 3b (ProstateX dataset) highlighted increased segmentation challenges. Several models failed to segment both zones simultaneously, leading to disconnected regions or missing zone labels. Additionally, models often produced asymmetrical or jagged boundaries that misrepresented prostate anatomy. SynESAM, on the other hand, maintained smooth contours, accurately preserved the spatial relationship between zones, and minimized deviations from expected anatomical shapes, even under conditions with poor contrast or image artifacts. These results underscore SynESAM’s robustness, adaptability, and ability to generalize across diverse and challenging imaging conditions.

The observed disparity in accuracy between the PZ and TZ segmentation results can be attributed to inherent anatomical and structural differences. The PZ is typically smaller in volume and exhibits more irregular and heterogeneous boundaries, which makes consistent delineation across patients more challenging. In contrast, the TZ is centrally located within the prostate and generally presents a more uniform and well-defined morphology, allowing models to achieve higher segmentation accuracy. These intrinsic anatomical variations likely account for the performance gap between the two zones.

### Individual module contributions to generalization

A key strength of SynESAM lies in the complementary contributions of its individual modules, each of which enhances generalization and robustness in distinct ways:

#### **Transformer stage**

By leveraging the SAM image encoder, the transformer stage captures long-range dependencies and global contextual information across the prostate MRI. This improves robustness to variable imaging conditions and low-contrast boundaries, particularly in the peripheral zone (PZ), where conventional CNNs often struggle.

#### **Bridge stage**

The nested skip connections and feature fusion in the bridge stage ensure that multi-scale contextual information is preserved when transitioning from transformer token embeddings to CNN feature maps. This mitigates information loss and facilitates consistent generalization across datasets with heterogeneous imaging protocols.

#### **CNN stage**

The U-Net–based decoder provides precise localization and boundary refinement by modeling local structural details. This is particularly critical for maintaining anatomically coherent boundaries in challenging cases, ensuring that the segmentation output remains clinically reliable.

#### **Final feature fusion**

The adaptive weighting and context-aware prioritization mechanism integrate transformer-derived global features with CNN-derived local features. This fusion dynamically emphasizes the most informative representations, thereby enhancing segmentation accuracy across both high-contrast and low-contrast regions.

#### **Continual learning modules (EWC/vEWC)**

The synchronous learning framework, enabled by EWC and vEWC, constrains critical parameters while allowing flexible adaptation to new datasets. These modules directly improve cross-institutional robustness by preserving previously learned knowledge and minimizing catastrophic forgetting.

### Cross-institutional generalizability and continual learning

**Fig. 4:**
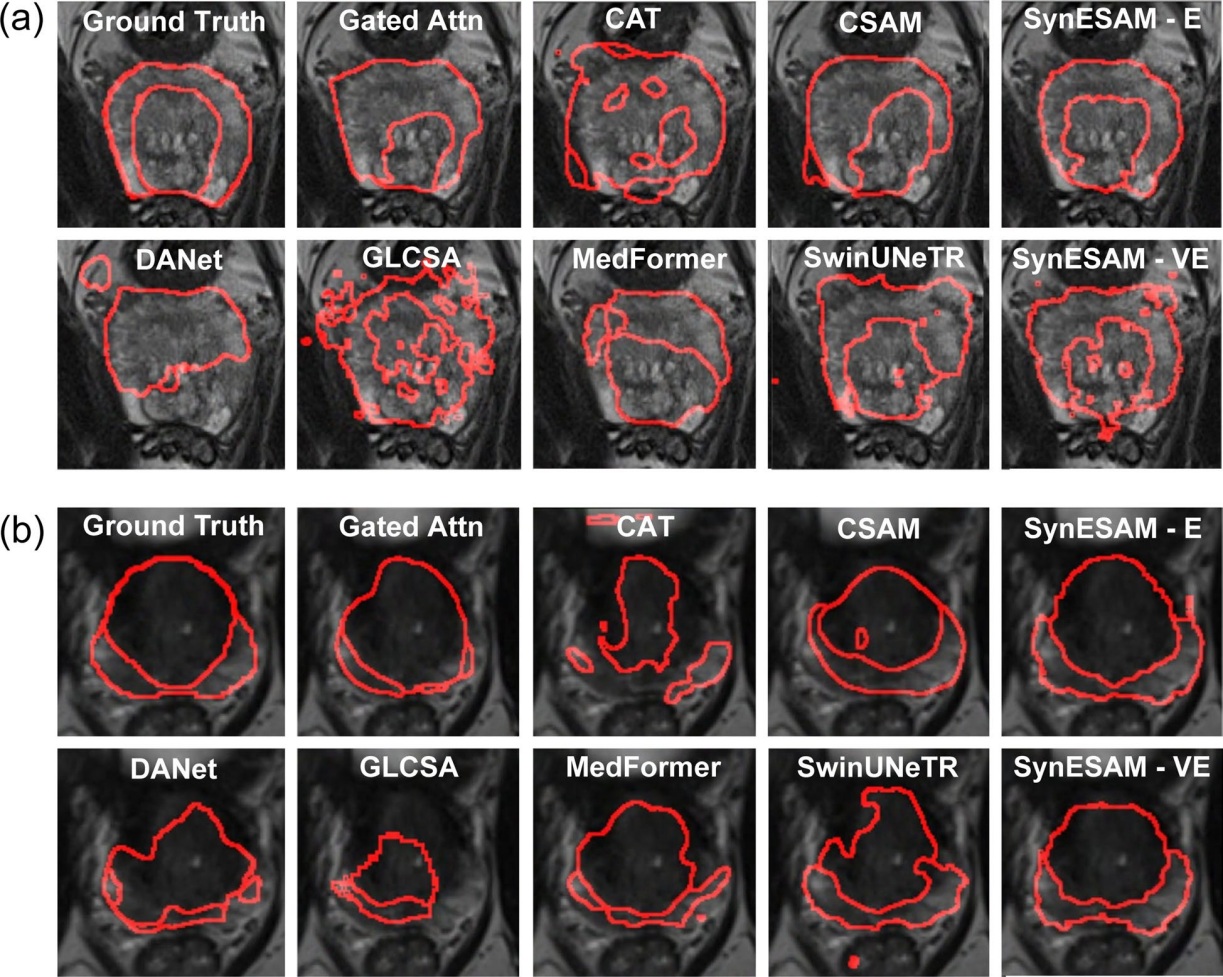
Representative prostate images with peripheral and transition zones determined by fine-tuned SynESAM using EWC and VEWC learning methods and seven other models. (a, b) Representative prostate images with the boundaries of peripheral and transition zones determined manually (ground truth) or by SynESAM using EWC (SynESAM-E), vEWC (SynESAM-VE), Gated Attn, CAT, CSAM, DANet, GLCSA, MedFormer, and SwinUNeTR, when (**a**) trained on UAB and fine-tuned on ProstateX or (**b**) trained on ProstateX and fine-tuned on UAB test datasets, were employed

Figure 4 evaluates how different models generalize across institutions through cross-dataset training and fine-tuning. Models were trained on one dataset and fine-tuned on another to simulate real-world domain shifts. SynESAM-E (with Elastic Weight Consolidation, EWC) and SynESAM-VE (with variational EWC, vEWC) stood out by delivering highly consistent and structurally reliable segmentations across institutions. In Fig. 4a (trained on UAB, fine-tuned on ProstateX), most models, including CAT, CSAM, and GLCSA, struggled with adaptation. These models produced shape distortions or misplaced contours due to rigidity or failure to adjust to domain-specific characteristics.

GLCSA, in particular, showed erratic boundary behavior and poor zone transitions. SynESAM-VE maintained structural stability while adapting, although with slight limitations in flexibility. SynESAM-E outperformed all others by integrating new domain features while retaining essential learned knowledge, thanks to EWC’s constraint-based regularization during learning. In Fig. 4b (trained on ProstateX and fine-tuned on UAB), the adaptation challenge became even more pronounced. CSAM, CAT, and MedFormer generated distorted segmentations that deviated from the true anatomy. GLCSA introduced artifacts that compromised boundary continuity. While SynESAM-VE showed strong retention of previously learned structures, it occasionally lagged in refining new boundaries. SynESAM-E again achieved the best balance, adjusting well to the new domain while maintaining overall segmentation accuracy, highlighting the value of EWC in multi-institutional learning scenarios.

### Stability and robustness: standard deviation (SD) and loss curve analysis

**Fig. 5:**
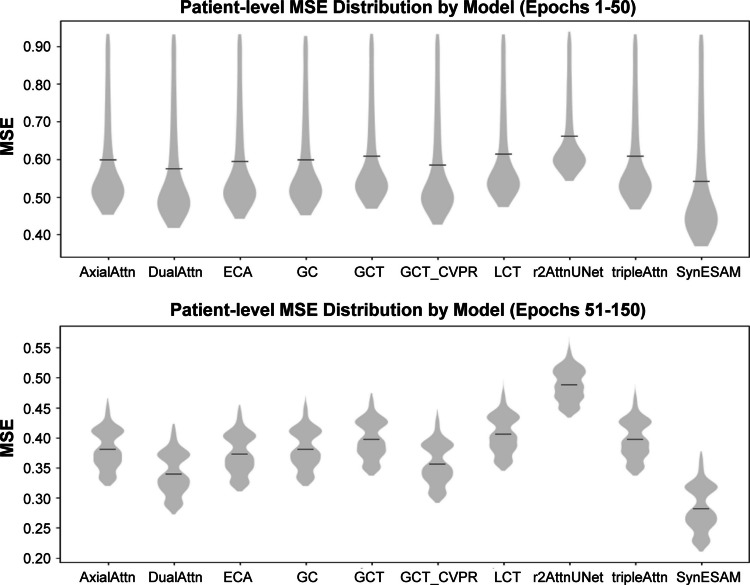
Patient-level training loss distributions (MSE) for different segmentation models from epochs 1 to 150. Each violin plot shows the distribution of mean squared error (MSE) across patients for a given model. SynESAM (Proposed) demonstrates consistently lower MSE values with tighter distributions compared to baseline models, reflecting both faster convergence and improved stability across patients

Beyond mean segmentation performance, standard deviation (SD) provides insight into the model’s consistency and robustness. A lower SD indicates stable and reliable performance across test samples. For the DSC and Mean Surface Distance (MSD), SynESAM generally produced lower SDs compared to other models. In particular, the SDs of the DSC were consistently lower in TZ than in PZ, reflecting TZ’s more regular structure and more precise boundaries. In contrast, the PZ showed higher variability across models due to its complex shape and low boundary contrast. SynESAM significantly reduced this variability, indicating its capacity to handle anatomical complexity effectively. For MSD, PZ also had a higher SD than TZ across models, reinforcing the challenges of PZ segmentation. Other models displayed even more variability in PZ segmentation, but SynESAM consistently lowered this deviation, offering greater segmentation reliability in anatomically complex regions. Figure 5 illustrates the training dynamics of all compared models, where we generated patient-level MSE loss curves over 150 epochs to assess convergence behavior and stability. The figure shows violin plots of patient-level distributions from epochs 1 and 150. As observed, SynESAM consistently achieved faster convergence and lower training loss compared to baseline transformers and CNN-based models. By epoch 50, SynESAM had already stabilized at substantially lower error levels, whereas competing methods continued to fluctuate. At later epochs, SynESAM maintained the lowest MSE with tight error distributions, reflecting both robustness and reduced variance across patients. In contrast, several baselines, such as r2 Attn UNet and tripleAttn, exhibited higher variance and slower convergence, highlighting their sensitivity to patient-level differences. These results underscore that SynESAM not only improves average performance but also delivers more stable and reproducible training outcomes across diverse patient data.

### Feature space analysis and knowledge retention

**Fig. 6:**
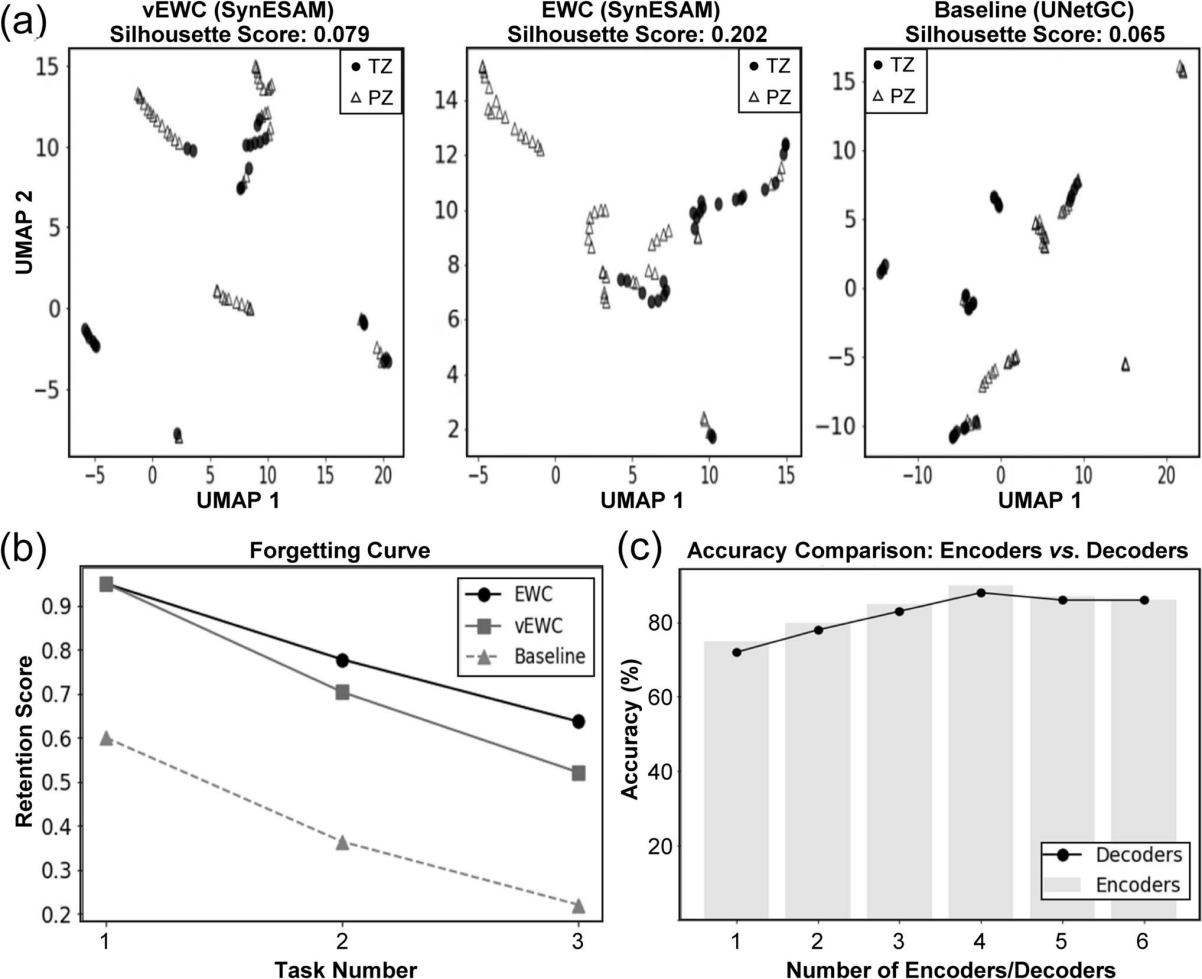
SynESAM model dynamics. (**a**) Class cluster feature representation when the proposed model is fine-tuned across various tasks using EWC and vEWC, as well as without any learning methods (baseline), to segment the peripheral (PZ) and transition zones (TZ). (**b**) The average knowledge retention scores of PZ and TZ segmentations across various tasks using EWC, vEWC, and without any learning methods (baseline). (**c**) Effect of the number of encoders and the decoders of the proposed model on the average accuracy

Uniform Manifold Approximation and Projection (UMAP) is a non-linear dimensionality reduction method that projects high-dimensional feature embeddings into a low-dimensional space while preserving both local and global data structure. It provides an intuitive way to visualize how well different anatomical zones are separated in the learned feature space. The Silhouette Score complements this by quantifying cluster separation, $s\left(i\right)=\frac{b\left(i\right)-a\left(i\right)}{\mathrm{max}\{a\left(i\right),b\left(i\right)\}}$, where $a\left(i\right)$ is the average intra-cluster distance and $b\left(i\right)$ is the nearest-cluster distance. Scores close to 1 indicate compact, well-separated clusters, while values near 0 suggest overlapping clusters. To assess knowledge retention and internal feature representation, UMAP visualizations of the first 30 UAB samples were generated and are presented in Fig. 6a. These visualizations, combined with Silhouette Scores, quantify the degree of cluster separation between the transitional zone (TZ) and peripheral zone (PZ) in the feature space after fine-tuning. The EWC-based model (SynESAM-E) achieved the highest Silhouette Score of 0.20, with clearly separated and compact clusters for TZ (circles) and PZ (triangles), reflecting strong knowledge preservation and minimal feature entanglement. The vEWC-based model (SynESAM-VE) yielded a lower score of 0.08, indicating slightly reduced cluster separation but still a reasonable balance between preserving prior knowledge and adapting to new data. In contrast, the baseline model without continual learning exhibited the lowest Silhouette Score of 0.07, with significant overlap between PZ and TZ clusters, evidence of catastrophic forgetting, and a marked degradation in the model’s ability to distinguish between anatomical zones.

### Forgetting curve analysis

Figure 6b illustrates the forgetting curves across a sequence of three tasks, with Task 3 centered on the Prostate158 dataset, which included 15 training and 20 testing samples. The forgetting curve quantifies the retention score, which reflects the model’s ability to preserve previously acquired knowledge after being exposed to new domains. SynESAM-E, utilizing EWC, demonstrated the most stable performance, with a gradual and minimal decline in retention across tasks, highlighting its effectiveness in constraining critical weights to mitigate forgetting. SynESAM-VE also preserved a substantial portion of prior knowledge, although its retention curve declined more steeply, suggesting a trade-off between adaptability and memory stability. In stark contrast, the baseline model showed a rapid deterioration in retention, retaining less than half of its original knowledge by Task 3. This sharp decline underscores the vulnerability of models without continual learning strategies to forget previously learned information when fine-tuned on new domains.

### Ablation study: SynDSAM vs. SynESAM

An ablation experiment was conducted to test an alternative model, SynDSAM, where the SAM image encoder was replaced with a ResNet50 backbone while retaining a modified SAM mask decoder that functions without explicit prompts. This hybrid was designed to evaluate whether strong convolutional features (via ResNet50) could match SynESAM’s performance. However, SynDSAM underperformed relative to SynESAM, particularly in terms of consistency in segmentation. On the UAB dataset, SynDSAM achieved a DSC of 0.86 for TZ and 0.65 for PZ, while on ProstateX, it reached 0.82 for TZ and 0.69 for PZ, both lower than SynESAM’s results, especially in PZ. The results highlight the importance of SAM’s image encoder, which captures rich global representations critical for accurate and fine-grained prostate zonal segmentation.

## Conclusion

In this study, we propose SynESAM, a hybrid Transformer-CNN architecture designed for automated prostate zonal segmentation, and demonstrate its superior accuracy and generalization across multi-institutional datasets. The model integrates the image encoder from the Segment Anything Model (SAM) with a CNN-based decoder and incorporates synchronous learning strategies, including Elastic Weight Consolidation (EWC) and its variant with reduced regularization strength (vEWC), to mitigate catastrophic forgetting during continual learning. This design enables SynESAM to maintain consistent performance across domains, a key requirement for clinical deployment in heterogeneous environments.

Accurate prostate zonal segmentation is central to multiple clinical tasks, including biopsy targeting, radiomic feature extraction, and personalized treatment planning. Compared to UNet-like conventional models, SynESAM offers two key advantages that increase its clinical reliability: first, its hybrid transformer, CNN design produces smoother, anatomically coherent boundaries that align more closely with expert radiologist annotations, particularly in challenging peripheral zone cases; second, its continual learning framework ensures that the model can be safely updated with new data from a different institution without erasing prior knowledge, thereby addressing a critical barrier to multi-center deployment. This ability to retain and transfer knowledge across diverse imaging settings reduces the risk of performance degradation under domain shift, a problem commonly faced in clinical translation. By delivering robust, fully automated segmentation that generalizes across centers, SynESAM has the potential to streamline diagnostic workflows, reduce reliance on labor-intensive manual delineation, and enable reliable adoption in multi-institutional prostate MRI practice. Future research will explore the integration of prompt-based semi-automatic strategies to enhance user interaction and clinical usability, as well as validate them on larger and more diverse cohorts to support further widespread clinical implementation.

## Data Availability

The UAB dataset utilized in this study is proprietary and was collected under an Institutional Review Board (IRB)-approved protocol at the University of Alabama at Birmingham. Due to patient privacy regulations and IRB restrictions, the UAB data cannot be publicly shared. The ProstateX dataset used in this study is publicly available and can be accessed at https://www.cancerimagingarchive.net/collection/prostatex/ as cited in the manuscript.

## Code availability

The source code developed for this study is made publicly available in the following GitHub repository: https://github.com/chetana348/SynSAM-for-Prostate-Segmentation.
